# Species Specificity of Type III Interferon Activity and Development of a Sensitive Luciferase-Based Bioassay for Quantitation of Mouse Interferon-λ

**DOI:** 10.1089/jir.2018.0066

**Published:** 2018-11-14

**Authors:** Sophie Jacobs, Fanny Wavreil, Bert Schepens, Hans Henrik Gad, Rune Hartmann, Joana Rocha-Pereira, Johan Neyts, Xavier Saelens, Thomas Michiels

**Affiliations:** ^1^ de Duve Institute, Université Catholique de Louvain, Brussels, Belgium.; ^2^ VIB Center for Medical Biotechnology, VIB, Ghent, Belgium.; ^3^ Department of Biomedical Molecular Biology, Ghent University, Ghent, Belgium.; ^4^ Department of Molecular Biology and Genetics, Aarhus University, Aarhus, Denmark.; ^5^ Laboratory of Virology and Chemotherapy, Department of Microbiology and Immunology, KU Leuven, Rega Institute for Medical Research, Leuven, Belgium.

**Keywords:** species-specific activity, cross-species activity, IFN-λ receptor, IFN-λ, type I IFN, antiviral, cytokine, respiratory syncytial virus

## Abstract

The type III interferon (IFN-λ) family includes 4 IFN-λ subtypes in man. In the mouse, only the genes coding for IFN-λ2 and -λ3 are present. Unlike mouse and human type I IFNs (IFN-α/β), which exhibit strong species specificity, type III IFNs were reported to act in a cross-specific manner. We reexamined the cross-specificity and observed that mouse and human IFN-λ exhibit some species specificity, although much less than type I IFNs. Mouse IFN-λ3 displayed clear species specificity, being 25-fold less active in human cells than the closely related mouse IFN-λ2. This specificity likely depends on amino acids in α helices A and F that diverged from other IFN-λ sequences. Human IFN-λ4, in contrast, retained high activity in mouse cells. We next developed a firefly luciferase-based reporter cell line, named Fawa-λ-luc, to detect IFN-λ in biological fluids with high specificity and sensitivity. Fawa-λ-luc cells, derived from mouse epithelial cells that are responsive to IFN-λ, were made nonresponsive to type I IFNs by inactivation of the *Ifnar2* gene and strongly responsive to IFN-λ by overexpression of the mouse IFNLR1. This bioassay was as sensitive as a commercially available enzyme-linked immunosorbent assay in detecting mouse IFN-λ in cell culture supernatant, as well as in serum and bronchoalveolar lavage samples of virus-infected mice. The assay also enabled the sensitive detection of human IFN-λ activity, including that of the divergent IFN-λ4 with a bias, however, due to variable activity of IFN-λ subtypes.

## Introduction

Type I and III interferons (IFNs) are typically produced in response to viral infection, and these cytokines induce an antiviral state in target cells (Isaacs and Lindenmann 1957; Kotenko and others 2003; Sheppard and others 2003). Type I IFNs consist of 13 IFN-α subtypes and IFN-β, -ω (human), and -ζ (mouse) -ɛ and -κ. The type III family of IFNs in humans comprises 3 functional genes that express IFN-λ1 (IL-29), IFN-λ2 (IL-28A), and IFN-λ3 (IL-28B) (Kotenko and others 2003; Sheppard and others 2003). A fourth IFN-λ subtype (IFN-λ4) is present in a part of the human population, depending on a dinucleotide frameshift polymorphism upstream of the *IFNL3* gene, which creates or disrupts the open reading frame (ORF) encoding IFN-λ4 (Prokunina-Olsson and others 2013). In the mouse, *Ifnl1* is a pseudogene, and only IFN-λ2 and IFN-λ3 are expressed. Unlike mouse and human type I IFNs that exhibit strongly species-specific activity (Veomett and Veomett 1979), type III IFNs were reported to act on cell types from both origins (Lasfar and others 2006; Hermant and others 2014).

The type I IFN family members signal through a unique heterodimeric receptor (IFNAR), composed of the IFNAR1 and IFNAR2 subunits. Type III IFNs engage a distinct receptor (IFNLR), composed of 2 chains: the IFN-λ-specific IFNLR1, and IL10Rβ which is shared by other IL10-related cytokines (Kotenko and others 2003; Sheppard and others 2003). While IFNAR is ubiquitously expressed, IFNLR is expressed by a restricted range of cell types and mostly acts at mucosal surfaces. Epithelial cells are well-established targets of IFN-λ *in vivo* (Sommereyns and others 2008), although some immune cells such as neutrophils and dendritic cells have been characterized as IFN-λ responders (Koltsida and others 2011; Blazek and others 2015; Broggi and others 2017; Espinosa and others 2017).

Although type I and type III IFNs act on distinct receptors, they activate a similar JAK-STAT transduction pathway, leading to the phosphorylation of STAT1 and STAT2 that associate with IRF9 to form the ISGF3 complex. Type I and type III IFN signaling leads to the transcription of an overlapping set of IFN-stimulated genes (ISGs) (Dumoutier and others 2004; Ank and others 2006).

Type I IFN in biological samples can be measured by a variety of bioassays, but efficient techniques for type III IFN quantification are largely lacking. Enzyme-linked immunosorbent assay (ELISA) for IFN-λ detection is time and cost intensive and fails to detect IFN-λ4. Conventional cytopathic effect reduction bioassays to quantify antiviral activity as a proxy for the presence of IFN require the manipulation of infectious virus. Luciferase reporter cells that have previously been used for recombinant human IFN-λ detection were still responsive to type I IFN and would not allow specific IFN-λ detection from biological samples (Uze and Monneron 2007). In this work, we reexamined the species specificity of IFN-λ activity, and we developed a very sensitive luciferase-based bioassay specific for type III IFN detection.

## Materials and Methods

### Cell culture

The LKR10 cell line (kind gift from Guido Bommer, de Duve Institute, Brussels, Belgium) is derived from lung adenocarcinoma tissues from a K-rasLA1 mouse (Johnson and others 2001). A549 cells (ATCC) were kindly provided by Pierre Coulie, BALB/3T3 fibroblasts (Aaronson and Todaro 1968) by Francis Brasseur (Ludwig Institute for Cancer Research, Brussels, Belgium), and Hela-M (Dong and others 2001) by Robert H. Silverman (Lerner Research Institute, Cleveland).

Those cells, and derivatives, were maintained in Dulbecco's modified Eagle's medium (DMEM) (Lonza, Vervier, Belgium) containing 4.5 g/L glucose, supplemented with 10% fetal calf serum (FCS) (Sigma-Aldrich, Overijse, Belgium). African green monkey kidney (Vero) cells (ATCC) were cultured in DMEM supplemented with 10% FCS, nonessential amino-acid, l-Glutamine, and sodium pyruvate. BHK-21 cells (ATCC) were cultured in Glasgow's minimum essential medium (Gibco; Thermo Fisher Scientific, Asse, Belgium) supplemented with 10% newborn calf serum and 2.95 g/L tryptose phosphate broth. All media were supplemented with 50 U/mL penicillin and 50 μg/mL streptomycin (Lonza).

### Vectors

Expression vectors used in this study are listed in Table 1. Plasmids pSJ1 and pSJ7 are pcDNA3 derivatives (Invitrogen; Thermo Fisher Scientific) used for mouse IFN-λ2 and IFN-λ3 expression in 293T cells. These plasmids were constructed by cloning the ORF encoding IFN-λ2 (pSJ1) and IFN-λ3 (pSJ7) between the *Bam*HI and *Xba*I sites of the vector. Coding sequences were polymerase chain reaction (PCR)-amplified from liver cDNA samples of a C57BL/6 mouse infected with influenza TURH (H7/N1) strain (Hermant and others 2014), using the following primers: Forward (Fw): *Bam*HI-*Age*I-Kozak-mIFN-λ2/3, 5′-AAA AGG ATC CAC CGG TGC CAC CAT GCT CCT CCT GCT GTT GCC TCT G-3′; Reverse (Rev): mIFN-λ2/3-stop-*Xba*I, 5′-AAA ATC TAG ATT ATC AGA CAC ACT GGT CTC CA**y** TGG C-3′.

**Table 1.** Expression Plasmids Used in This Study

*Vectors*	*Protein expressed, particularity*	*Type (parental vector)*
CRISPR-Cas9 nickase expressing vectors		
pSJ37	sgRNA 1-exon 6 mouse IFNAR2c	Plasmid (px461)
pSJ38	sgRNA 2-exon 6 mouse IFNAR2c	Plasmid (px461)
IFN-α expression plasmids		
pcDNA3-IFNαA	IFNαA-mouse	Plasmid (pcDNA3)
pPH23	IFNα2-human	Plasmid (pcDNA3)
IFN-λ expression plasmids		
pSJ1	IFN-λ2-mouse (C57bl/6 genomic sequence)	Plasmid (pcDNA3)
pSJ7	IFN-λ3-mouse (C57bl/6 genomic sequence)	Plasmid (pcDNA3)
pCS59	IFN-λ3-mouse (pEF-2-mIFN-λ3 derivative)	Plasmid (pcDNA3)
Lentiviral constructs		
pMK03	Mx1-promoter-*Firefly* luciferase	Lentiviral (pCCL)
pSJ12	IFNLR1 (IRES-mCherry)	Lentiviral (pTM945)
Empty vector		
pTM945	IRES-mCherry	Lentiviral (pCCL)

CRISPR, clustered regularly interspaced short palindromic repeats; IRES, internal ribosome entry site.

The sequence of pSJ7 matches the genomic *Ifnl3* sequence but diverges from a few nucleotides from that of the previously constructed pCS59 plasmid (Sommereyns and others 2008). Both plasmids encode functional IFN-λ3. pPH23 was obtained by PCR-cloning the *IFNA2* ORF from Hela-M cell cDNA, between the *Eco*RI and *Not*I sites of pcDNA3 (Fw: *Eco*RI-Kozak-IFNα2, 5′-AAA AGA ATT CAC CAT GGC CTT GAC CTT TGC TTT-3′; Rev: IFNα2-*Not*I, 5′-AAA AGC GGC CGC TCA TTC CTT ACT TCT TAA ACT TT-3′).

pMK03 is a lentiviral vector, derived from pTM897, that carries the mouse *Mx1* gene promoter driving the expression of the firefly luciferase gene. It is derived from pCCLsin.cPPT.hPGK.GFP.pre (Follenzi and others 2000) in which a cloning polysite was first inserted to replace the coding sequences, yielding pTM897. The firefly luciferase gene from pGL4.16 (Promega) was inserted between the *Xho*I and *Xba*I sites of the vector, and the *Mx1* promoter was then cloned from pBSK-Mx1, a gift from Peter Staeheli (Freiburg University, Freiburg, Germany), as a *Bam*HI/*Bsa*BI fragment.

The lentiviral vector pSJ12, used for expression of the mouse IFN-λ receptor, was obtained by cloning the *Ifnlr1* ORF between the *Bam*HI and *Xba*I sites of pTM945, a lentiviral vector allowing the coexpression of the cloned gene and of mCherry (Hermant and others 2013). The *Ifnlr1* ORF sequence was subcloned in this plasmid from pcr2.1-LICR2, kindly provided by Laure Dumoutier (de Duve Institute, Brussels, Belgium).

### Clustered regularly interspaced short palindromic repeats Cas 9 editing

Gene inactivation was done with pX461 plasmid (pSpCas9n-2A-GFP) coding for the Cas9 nickase and green fluorescent protein (GFP) (Ran and others 2013a). The 2 single guide RNAs (sgRNAs) were designed using the MIT clustered regularly interspaced short palindromic repeats (CRISPR) design tool website (http://crispr.mit.edu). The selected sgRNA pair (sgRNA1- Fw: 5′-CAC CGT CAA ATT CTG GCG GCT CAA G-3′; Rev: 5′-AAA CCT TGA GCC GCC AGA ATT TGA C-3′; sgRNA2- Fw: 5′-CAC CGA GAC CAC ATA AAC GTG ACG A-3′; Rev: 5′-AAA CTC GTC ACG TTT ATG TGG TCT C-3′) was targeting exon 6 of the mouse *Ifnar2* gene (GenBank: Y09865.1) and exhibited no expected off-target cleavage site.

The sgRNAs were cloned into pX461 to form the plasmids pSJ37 and pSJ38. These constructs were cotransfected in LKR10 cells, using *Trans*IT^®^-LT1 transfection reagent (Mirus Bio LLC, Madison), according to the manufacturer's instructions. After 48 h, GFP-positive cells were sorted by fluorescence activated cell sorting (FACS Aria III; BD Biosciences) and cloned in 96-well plates. Clones were screened for loss of type I IFN response with an antiviral assay. Genome editing of the targeted exon was further confirmed by sequencing (Genewiz).

### ISG induction and quantification

ISG expression was measured by reverse transcription-quantitative PCR. IFNAR2-knockout (KO) and wild-type (WT) LKR10 cells were treated with 100 U/mL mouse IFN-αA, 700 pg/mL mouse IFN-λ3, or control supernatant (mock) for 8 h before RNA extraction. Total RNA extraction, reverse transcription, and SYBR Green quantitative PCR for mRNA encoding mouse β-actin, Oasl2, and Usp18 were performed as previously described (Paul and Michiels 2006).

### Flow cytometry

For infection analysis, cells were dissociated with trypsin-EDTA and suspended in phosphate-buffered saline (PBS) containing 5% of filtered FCS and 0.5% of paraformaldehyde. Data acquisition was done with an LSRFortessa flow cytometer (BD Biosciences) using FACSDiva software. Data were analyzed using FlowJo 9.6.4. The rate of infection was defined as the percentage of mCherry-positive cells 24 h postinfection with 0.5 PFU/cell TM967.

For cell sorting, transduced cells were suspended in PBS containing 1% FCS and 1 mM EDTA. mCherry- or GFP-positive cells were cloned at 1 cell per well in 96-well plates using the FACS Aria III (BD Biosciences).

### Immunoblotting

STAT1, P-STAT1, and β-actin were detected by Western blot using sodium dodecyl sulfate–polyacrylamide gel electrophoresis gels containing 10% acrylamide and run in a Tris-Glycine buffer. Blots were probed with anti-STAT1 polyclonal (sc-346; Santa Cruz Biotechnology, Heidelberg, Germany), P-STAT1 monoclonal (#9167; Cell Signaling Technology, Leiden, The Netherlands), and anti-β-actin monoclonal (A5441; Sigma-Aldrich) antibodies.

### IFNs

Mouse IFN-αA, IFN-β, IFN-λ2, IFN-λ3, and human IFN-α2 were produced as described previously from 293T cells transfected with pcDNA3-IFN-αA, pcDNA3-IFN-β (van Pesch and others 2004), pcDNA3-IFN-λ2 (pSJ1), and pcDNA3-IFN-λ3 (pSJ7). Human IFN-α2 was produced similarly, using pcDNA3-IFN-α2 (pPH23). Supernatant collected from 293T cells transfected in parallel with the empty pcDNA3 vector was used for control treatment (mock) of cells and diluted as for IFNs.

Recombinant mouse IFN-λ3 (1789-ML-025) and human IFN-λ1 (1598-IL-025), IFN-λ2 (1587-IL-025), and IFN-λ3 (5259-IL-025) were purchased from R&D (R&D Systems, Minneapolis). Recombinant human IFN-λ4 was produced as described (Hamming and others 2013). Mouse (ref 315-05) and human (ref 300-02) IFN-γ were purchased from PeproTech (London, United Kingdom). GenBank accession numbers for mouse and human IFN-λ are listed in Table 2.

**Table 2.** GenBank Accession Numbers for Interferon-λ Sequences

	*Nucleotide*	*Protein*
mIFN-λ2	NM_001024673.2	NP_001019844.2
mIFN-λ3	NM_177396.1	NP_796370.1
huIFN-λ1	NM_172140.1	Q8IU54.1
huIFN-λ2	NM_172138.1	NP_742150.1
huIFN-λ3	NM_001346937.1	AAN28264.1
huIFN-λ4	NM_001276254.2	NP_001263183.2

### IFN quantification assays

IFNs were diluted in culture medium for cell treatment or in reagent diluent for ELISA. Mouse IFN-λ2 and IFN-λ3 were quantified by ELISA. Mouse IFN-αA and human IFN-α2 antiviral activities were quantified, as described previously, by a cytopathic effect reduction assay in mouse BALB-3T3 and human Hela-M cells, respectively, using Mengovirus (van Pesch and Michiels 2003). Similar assays were conducted in human A549 cells and mouse LKR10 cells for quantification of IFN-λ cross-species activity. IFN-λ4 was quantified by a cytopathic effect reduction assay in A549 cells, using recombinant human IFN-λ3 as a standard, which was reported to have a similar specific activity (Hamming and others 2013).

### IFN-λ2/3 ELISA

Mouse IFN-λ2/3 measurement by ELISA was performed using the mouse IFN-λ2/3 DuoSet (R&D Systems), according to the manufacturer's protocol. Mouse sera were diluted 5- to 10-fold, and bronchoalveolar lavage fluids (BALFs) were diluted 5-fold.

### Ultraviolet light irradiation

When required (in case of samples derived from infected cells or mice), infectious virus present in biological samples was ultraviolet light (UV)-inactivated. For UV inactivation, 2.5 mm thick fluid samples were exposed at fixed UV dose (0.25–0.5–1–2 J/cm^2^) with the UV irradiation system Bio-link 254 (Vilber Lourmat, Eberhardzell, Germany), either in 24- (470 μL) or in 96-well (80 μL) plate. UV-exposed samples were titrated on BHK-21 cells by a standard plaque assay to confirm virus inactivation.

To confirm that the UV irradiation procedure that was established for picornaviruses effectively inactivates respiratory syncytial virus (RSV), 3 samples of 470 μL containing 1.5 × 10^6^ PFU of RSV were exposed to 2 J/cm^2^ with the UV irradiation system Bio-link 254 at room temperature in a 24-well plate (UV irradiated samples). As controls 3 similar samples of 470 μL containing 1.5 × 10^6^ PFU of RSV were incubated at room temperature in a 24-well plate (untreated samples). The titers of replicating RSV in the UV irradiated and untreated samples were determined by plaque assay using Vero cells.

### IFN luciferase assay

Cells were seeded at 160,000 cells per well in 24-well plates for cell supernatant and recombinant IFN analysis or at 32,000 cells per well in 96-well plates for mouse serum and BAL analysis. Forty-eight hours after seeding, cells were treated with IFN-λ2/3 or mock-treated or incubated with UV-treated mouse serum (50- and 100-fold dilutions) or BAL (5-fold dilutions). Samples were diluted in culture medium. Recombinant mouse IFN-λ3 provided in the ELISA Kit was used as a standard.

Firefly luciferase activity was measured 6 h after treatment using the luciferase assay system (Promega). Twenty microliters and 100-μL lysis buffer were used in 96- and 24-well plates, respectively; 10 μL of the lysate was mixed with 25 μL of substrate for detection. Luminescence was measured with a GloMax 20/20 luminometer (Promega). The limit of detection (LOD) for each test was established based on the mean of mock-treated samples, plus 3 standard deviations, in all assays.

### Viruses

TM967 is a Theiler's murine encephalomyelitis virus (TMEV) (strain DA) derivative that carries a capsid adapted to infect L929 cells and the ORF encoding the mCherry fluorescent protein cloned as a *Xba*I*/BsiW*I fragment to replace codons 5–67 of the leader protein coding region, in the pKJ6 vector (Jnaoui and Michiels 1998). Mengovirus (a strain of encephalomyocarditis virus) used for IFN bioassay is an attenuated variant that has been described previously (Hermant and others 2013). Those viruses were quantified by plaque assay on BHK21 cells.

RSV propagation and enrichment were performed as described in Schepens and others (Schepens and others 2014).

### Mice and viral infection

Mouse experiments were conducted according to the national (Belgian Law 14/08/1986 and 22/12/2003, Belgian Royal Decree 06/04/2010) and European (EU Directives 2010/63/EU, 86/609/EEG) animal regulations. Animal protocols were approved by the Ethics Committee of Ghent University (permit no. LA1400091, approval ID 2013-030). All efforts were made to avoid or ameliorate suffering of animals.

Specific pathogen-free female BALB/c mice at the age of 7–8 weeks were purchased from Charles River. The mice were housed in a specific-pathogen-free temperature controlled environment with 12-h light/12-h dark cycles and given water and food *ad libitum*. Mice were used at 9 weeks of age after adaptation in the animal room for 1 week. For challenge, the mice were sedated with isoflurane and infected intranasally with 4 × 10^6^ PFU of human RSV. Mock infection of the control group was performed with Hank's balanced salt solution.

Five days postinfection, BAL was isolated under anesthesia with an intraperitoneal injection of avertin (2.5% in PBS). A 23-gauge cannula was inserted into the trachea, and cells were collected by washing the airway lumen twice with 0.5 mL PBS containing 0.05 mM EDTA. After removing the cells by centrifugation (5 min at 400*g*), the BALF was stored at −80°C.

Serum samples from norovirus-infected and plasmid-electroinjected mice were reused from a previously published experiment (Rocha-Pereira and others 2018) to minimize animal experimentation.

### Statistics

Statistical analysis was performed using Prism 7 software (GraphPad Software, San Diego, CA). [***P < 0.001, **P < 0.01, *P < 0.05, and ns no statistically significant difference (*P* ≥ 0.05)].

## Results

### Species-specific activity of mouse and human IFN-λ

Unlike mouse and human type I IFNs (IFN-α/β) that exhibit strong species specificity, type III IFNs were reported to act in a cross-species manner. To confirm whether a mouse cell-based bioassay would permit the detection of IFN-λ subtypes from 2 different mammalian species, the species specificity of mouse and human type III IFNs was reexamined. Cross-reactive antiviral activity was systematically compared in a cytopathic effect reduction assay, using epithelial cell lines from mouse (LKR10) and human (A549) origin.

**FIG. 1.**
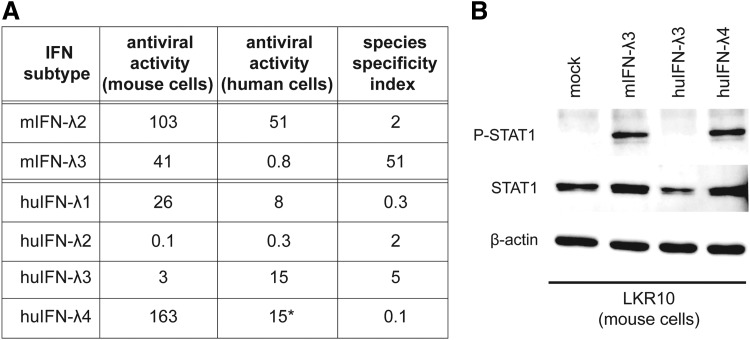
Species specificity of mouse and human type III IFNs. **(A)** Table showing the relative antiviral activity of mouse and human IFN-λ on mouse LKR10 and human A549 cells. Species specificity index was calculated as the ratio between the relative antiviral activity on cells of homologous to nonhomologous species (eg, for mouse IFN: relative activity in mouse cells/relative activity in human cells). Relative activities in a given cell line were calculated as the IFN dilutions (starting from 1 ng/mL) that yielded similar antiviral activities. *Due to the low amount of IFN-λ4 available, the initial concentration of this IFN was estimated by comparison with human IFN-λ3 antiviral activity that was reported to have a similar specific activity. **(B)** Western blot showing STAT1 phosphorylation in mouse LKR10 cells 30 min after treatment with control medium (mock) or IFN-λ. Concentrations of human (Hu) IFN-λ3 (615 pg/mL) and IFN-λ4 that yielded the same antiviral activity on human A549 cells were used. As a control, mouse IFN-λ3 was used at a concentration (2.5 ng/mL) showing equivalent antiviral activity as human IFN-λ4 on mouse cells. Results are representative of 3 experiments.

As expected, type I human (IFN-α2) and mouse (IFN-αA) IFNs were extremely species-specific in the antiviral assay, with an activity difference higher than 10,000-fold when applied to mouse and human cells. IFN-λ exhibited some level of species specificity although much less than type I IFNs (Fig. 1A). Mouse IFN-λ3 displayed the most pronounced species specificity. For a quantity that yielded the same antiviral activity in mouse cells, IFN-λ3 was 25 times less active in human cells than the closely related mouse IFN-λ2. Human IFN-λ1, IFNλ-2 and, to a lesser extent, IFNλ-3 exhibited significant antiviral effect on mouse epithelial cells.

As IFN-λ4 has been shown to have a specific activity similar to that of human IFN-λ3 in human cells, it was quantified accordingly using the antiviral assay (Hamming and others 2013). Remarkably, human IFN-λ4 displayed much more antiviral activity on mouse cells than equivalent amounts of human IFN-λ3. Accordingly, treatment of LKR10 cells with concentrations of human IFN-λ3 and IFN-λ4 that yielded equivalent antiviral activities in human cells resulted in clear STAT1 phosphorylation after IFN-λ4 but not after IFN-λ3 treatment (Fig. 1B).

### Generation of IFNAR2 knockout LKR10 cells

**FIG. 2.**
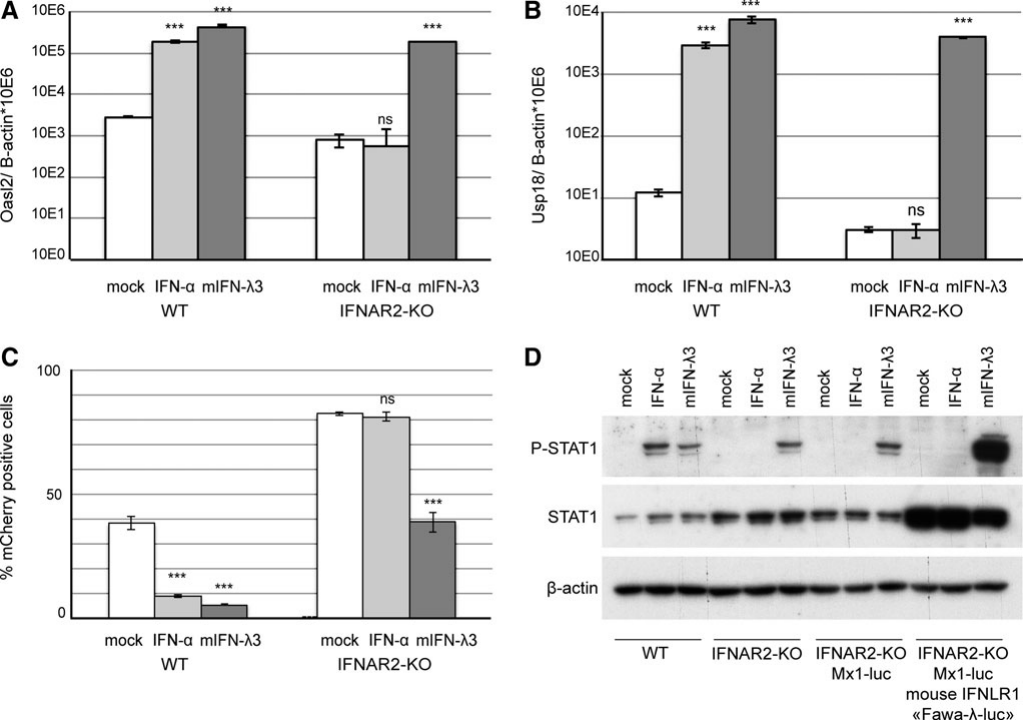
IFN-stimulated gene expression, STAT1 phosphorylation, and antiviral activity in response to IFN-αA and IFN-λ3 treatment in LKR10 derived cells. **(A, B)** Amounts of *Oasl2* **(A)** and *Usp18* **(B)** transcripts per 10^6^ *β-actin* copies detected in WT and IFNAR2-KO LKR10 cells 24 h after treatment with mouse IFN-αA, IFN-λ3 (mIFN-λ3), or control medium (mock). **(C)** Percentage of mCherry-positive cells in WT and IFNAR2-KO LKR10 cells measured by flow cytometry 24 h postinfection with 0.5 PFU/cell TM967. Cells were pretreated with mouse IFN-αA, mIFN-λ3, or mock for 7 h before infection. **(D)** Western blot showing STAT1 phosphorylation and expression in LKR10 cells and derivatives. Cells were treated for 30 min before protein extraction. Reproducible results were obtained in 3 independent experiments. **(A–D)** 100 U/mL IFN-αA and 700 pg/mL mIFN-λ3 were used for treatment. Student's *t*-test: ***indicates a significant difference of the IFN-treated groups compared to the mock-treated group **(A–C)**. ns, nonsignificant. WT, wild-type.

In line with their epithelial origin, mouse LKR10 cells respond to IFN-λ. To ensure a selective detection of type III IFNs, the gene coding for the IFNAR2 subunit of the type I IFN receptor was inactivated using the double nickase CRISPR/CRISPR-associated (Cas) 9 technology (Ran and others 2013b). An LKR10-IFNAR2-KO clone was selected for the absence of response to type I IFN and intact response to type III IFN. Sequencing of the targeted exon revealed frameshift mutations in both *Ifnar2* alleles, resulting in premature STOP codon appearance. In contrast to IFN-λ treatment, IFN-α treatment of this clone failed to induce the expression of the IFN-stimulated genes, *Oasl2* (Fig. 2A) and *Usp18* (Fig. 2B), and to protect against infection with TM967, a TMEV derivative expressing mCherry (Fig. 2C).

### Generation and characterization of Mx1-luciferase reporter cells for type III IFN detection

To develop IFN-λ reporter cells, the LKR10-IFNAR2-KO clone was transduced with a lentiviral vector encoding the firefly luciferase gene under the control of the *Mx1* promoter. Among the clones tested for luciferase activity induction after IFN-λ treatment, the best one displayed not more than 10-fold luciferase activity induction. To try to boost the reporter gene responsiveness to IFN-λ, this clone was transduced with SJ12, a lentiviral vector that coexpresses the mouse IFNLR1 and mCherry. Transduced cells were sorted, based on mCherry expression, and cloned.

**FIG. 3.**
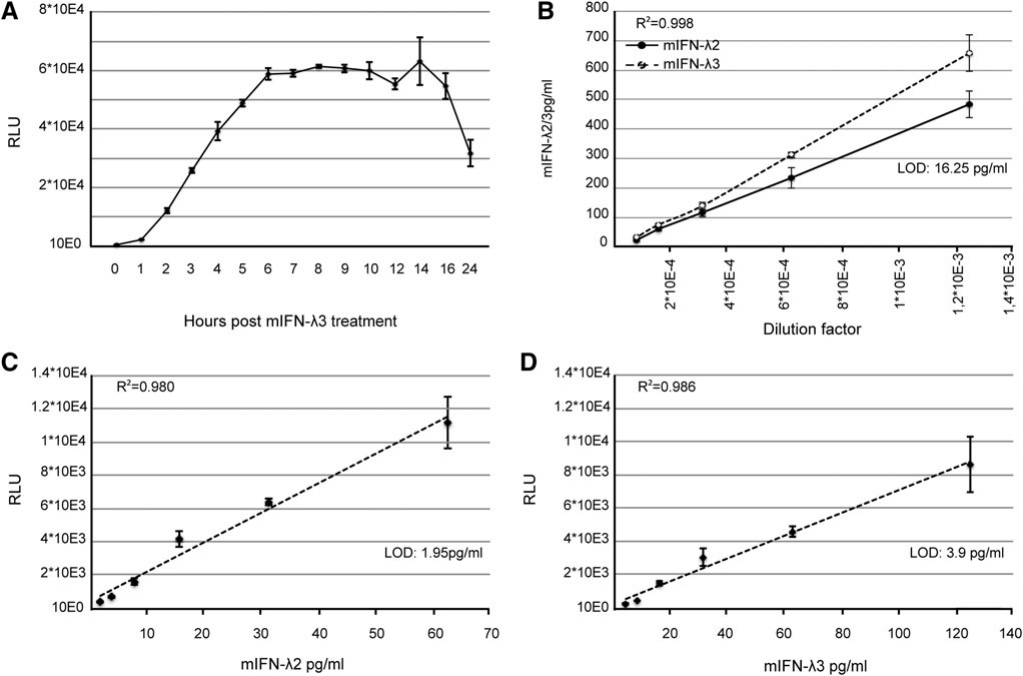
Mouse IFN-λ titration using Fawa-λ-luc reporter cells and ELISA. **(A**) Luciferase activity detected in Fawa-λ-luc cells treated for the indicated time with 700 pg/mL mouse IFN-λ3 (mIFN-λ3). **(B)** Quantification by ELISA of mouse IFN-λ2 (mIFN-λ2) and mIFN-λ3 in cell supernatants. Two fold serial dilutions (800–12,800-fold) were quantified in quintuplicate. **(C, D)** Dose–response of mIFN-λ2 and mIFN-λ3 supernatants was measured in triplicate Fawa-λ-luc cells and is representative of at least 3 independent experiments. Cells were treated for 6 h with 2-fold serial dilutions. Data points in the linear range of the assays were *plotted*, and linear regression analysis was performed. LOD is based on the mean of mock treated cell signal, plus 3 standard deviations. ELISA, enzyme-linked immunosorbent assay; LOD, limit of detection; RLU, relative light units; R^2^, coefficient of regression.

A selected clone, named “Fawa-λ-luc,” responded vigorously to IFN-λ3. This clone displayed increased constitutive STAT1 expression and strong STAT1 phosphorylation after IFN-λ3 but not after IFN-α treatment (Fig. 2D). A significant luciferase activity increase was measured in Fawa-λ-luc cells as soon as 1 h after treatment with 700 pg/mL (quantified by ELISA) of mouse IFN-λ3. Maximal induction was reached within 6 h and lasted up to 16 h post-treatment (Fig. 3A).

### Fawa-λ-luc cells allow sensitive detection of mouse IFN-λ2 and -3

The sensitivity of the Fawa-λ-luc assay was compared to that of an available ELISA test for mouse IFN-λ. Mouse IFN-λ2 and -3 produced by transfected 293T cells and recombinant mouse IFN-λ3 were quantified in parallel by ELISA and the Fawa-λ-luc assay. In the ELISA, responses were linear between 1,000 and 16.25 pg/mL, the experimental LOD of this test (Fig. 3B). In the Fawa-λ-luc assay, linearity was reached in a narrower concentration range (1.95–62.5 pg/mL for mouse IFN-λ2; 3.9–125 pg/mL for mouse IFN-λ3), but a higher sensitivity was observed with the detection limit being below 1.95 pg/mL and 3.9 pg/mL, for IFN-λ2 and -3, respectively (Fig. 3C, D). Based on ELISA quantification, mouse IFN-λ2 bioactivity in this assay was 2-fold higher than that of mouse IFN-λ3.

Importantly, after up to 29 passages, the intensity of the luciferase signal in the Fawa-λ-luc cells tended to decrease, but the sensitivity of the bioassay was preserved because the background activity also decreased with high passage numbers.

To confirm the specificity of Fawa-λ-luc cells for IFN-λ, we also evaluated any possible luciferase induction after treatment with type I and type II (ie, IFN-γ) IFNs. As expected, no signal was observed after treatment with up to 2,500 IU/mL of either mouse IFN-αA or IFN-β. Human IFN-γ also failed to induce a detectable luminescent signal in the Fawa-λ-luc cells in the tested concentration range (0–25 ng/mL). The LOD for mouse IFN-γ was 12.55 ng/mL, a concentration at least 10-fold higher than what is observed in mouse fluids in infectious contexts (Pomeroy and others 1998; Claser and others 2017). IFN-γ is thus not expected to interfere with IFN-λ detection in biological samples.

### Detection of human IFN-λ subtypes

**FIG. 4.**
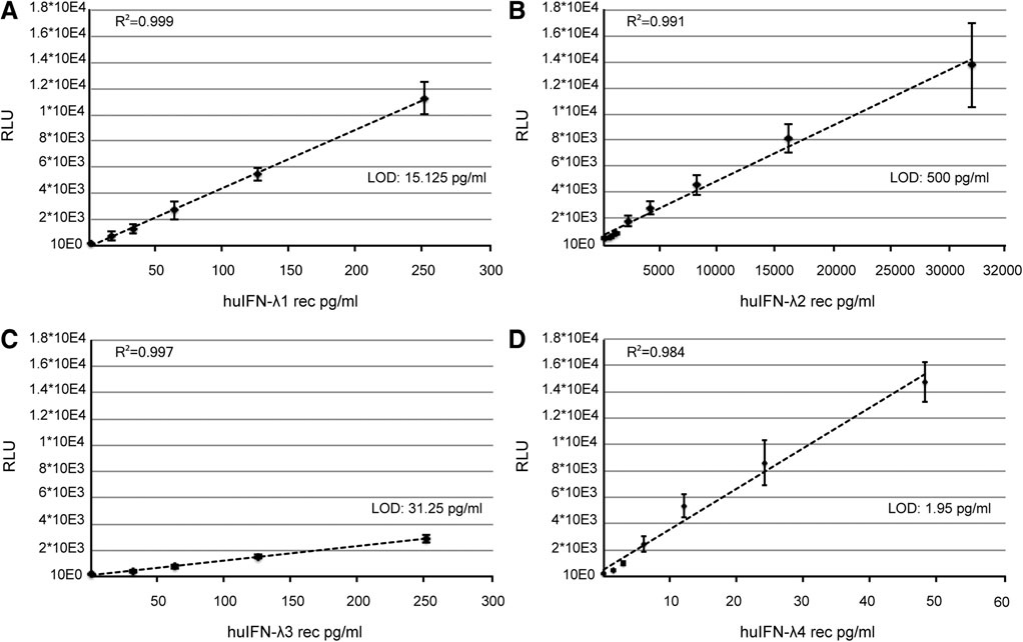
Human IFN-λ response in the mouse Fawa-λ-luc reporter cells. **(A–D)** Dose–response of human (hu) recombinant **(A)** IFN-λ1, **(B)** IFN-λ2, **(C)** IFN-λ3, and **(D)** IFN-λ4 as measured in triplicate. **(A–D)** Data points in the linear range of the assays were *plotted*, and linear regression analysis was performed. LOD is based on the mean of mock treated cell signal, plus 3 standard deviations.

We tested the sensitivity of our bioassay for human IFN-λ detection. Responses were analyzed individually for all 4 human IFN-λ subtypes (Fig. 4A–D). As their cross-species activity differed, different detection sensitivities were reached with our mouse cell-based reporter assay: 15.125 pg/mL for IFN-λ1, 500 pg/mL for IFN-λ2, 31.25 pg/mL for IFN-λ3, and 1.95 pg/mL for IFN-λ4. The bioassay turned out to be extremely sensitive for human IFN-λ4 detection. It is, however, worth noting that the recombinant IFN-λ4 stock concentration was determined by comparison of its activity with that of a human IFN-λ3 standard.

### Ultraviolet light resistance of IFN-λ

When testing biological samples, potential occurrence of infectious virus may interfere with the viability of reporter cells. It is therefore important to inactivate infectious viruses in such samples. As IFN-λ had previously been shown to be acid labile (Kugel and others 2011), UV inactivation of the virus was preferred. We thus tested whether virucidal UV doses would affect IFN-λ activity. To this end, TM967 virus, mouse IFN-λ2, and mouse IFN-λ3 were irradiated with increasing UV doses. Mock- and UV-exposed samples were then analyzed with the bioassay for mouse IFN-λ2 and IFN-λ3 detection and by plaque assay to determine viral titers.

**FIG. 5.**
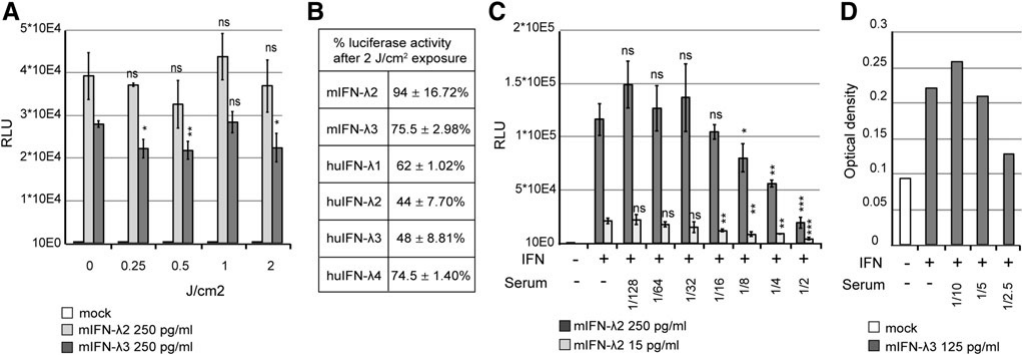
Sensitivity of IFN-λ to UV exposure and influence of serum on reporter cell activity and ELISA detection. **(A, B)** UV sensitivity of mouse and human IFN-λ. **(A)** Relative luciferase activities of Fawa-λ-luc cells treated with 250 pg/mL of mouse IFN-λ2 (mIFN-λ2) or IFN-λ3 (mIFN-λ3) supernatants irradiated (*n* = 3) under 0.25, 0.5, 1 and 2 J/cm^2^. **(B)** Percentage of IFN-λ activity (mean and SD) remaining after UV treatment (*n* = 3) at 2 J/cm^2^. IFN activity was measured in Fawa-λ-luc cells for IFN concentrations that yielded equivalent luciferase activities (20,000 RLU) before UV treatment (250 pg/mL mIFN-λ2 and mIFN-λ3, 125 pg/mL human IFN-λ1, 10 ng/mL huIFN-λ2, 500 pg/mL huIFN-λ3, and 62.5 pg/mL huIFN-λ4). **(C, D)** Influence of serum dilution on IFN-λ detection by Fawa-λ-luc cells **(C)** and ELISA **(D)**. **(C)** Fawa-λ-luc cells were treated in triplicate with fixed doses of 15 and 250 pg/mL mIFN-λ2 supernatant and with 2-fold serial dilutions (2–128-fold) of control mouse serum. **(D)** 125 pg/mL recombinant mIFN-λ3 was mixed with 2-fold serial dilutions of control mouse serum (2.5–10-fold) before detection by ELISA. **(A–C)** Reporter cells were exposed to IFN for 6 h before luciferase assay. Student's *t*-test: */**/***denotes a significant difference in signal compared to no UV exposure **(A)** or the absence of serum **(C)**. UV, ultraviolet light.

A 2 J/cm^2^ UV exposure was sufficient to ensure a virus titer reduction of more than 100,000-fold (5.5 × 10^5^ PFU to <1 PFU). At this UV dose, mouse IFN-λ2 activity was not significantly reduced and mouse IFN-λ3 showed a bioactivity reduction of less than 25% (Fig. 5A).

Similarly, IFN-λ1 and IFN-λ4 bioactivities were reduced by 25%, while human IFN-λ2 and IFNλ-3 were slightly more sensitive to irradiation, with about 2-fold activity decrease (Fig. 5B).

### Highly sensitive detection of IFN-λ2/3 in mouse serum and bronchoalveolar lavage fluids

We then compared the sensitivities of the Fawa-λ-luc and of the ELISA assays to detect IFN-λ in mouse serum and BAL samples. IFN-λ was first quantified in the serum of mice that were either injected intramuscularly with an IFN-λ3 expressing plasmid (pCS59), thus expected to produce circulating IFN-λ, or infected with mouse norovirus (Rocha-Pereira and others 2018).

**FIG. 6.**
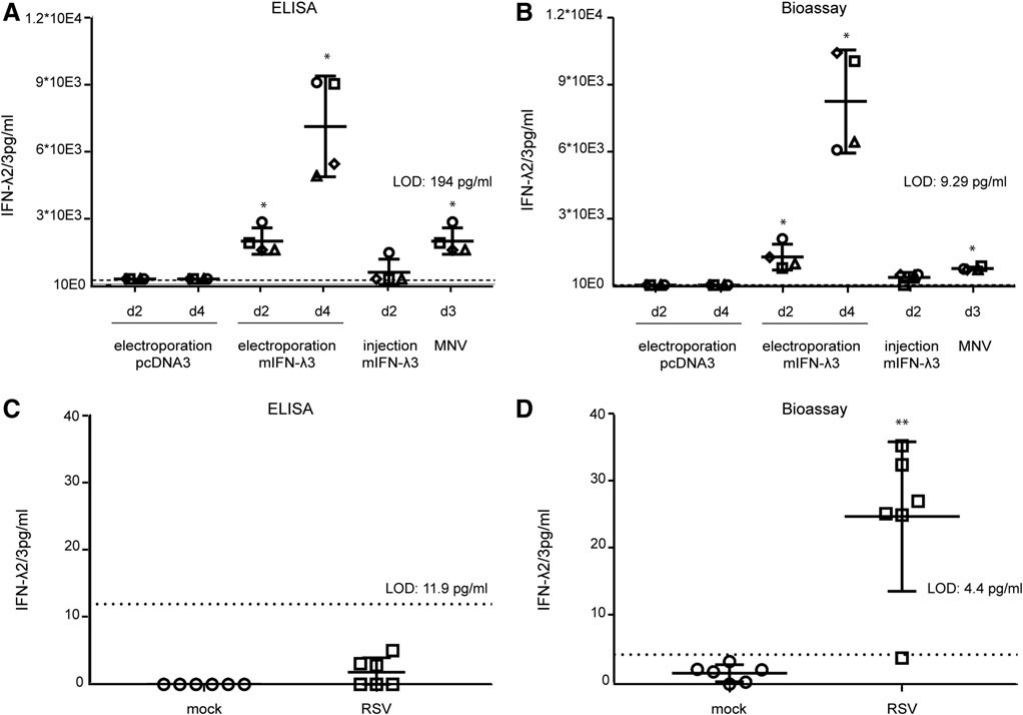
Detection of type III IFN in biological samples by ELISA and bioassay. **(A, B)** IFN-λ2/3 detection by ELISA **(A)** and bioassay **(B)** in the serum of AG129 mice 2 or 4 days after electroinjection of mouse IFN-λ3 (mIFN-λ3) expressing (pCS59) or empty (pcDNA3) plasmids, 2 days after injection of pCS59, or 3 days after infection with mouse norovirus. IFN-λ detection in the serum by ELISA was performed without UV exposure to keep maximal sensitivity. **(C, D)** IFN-λ2/3 detection by ELISA **(C)** and bioassay **(D)** in the bronchoalveolar lavage of BALB/C mice, 5 days postinfection with RSV, compared to control mice (mock). BALF were UV-exposed before testing. **(A–D)** The horizontal *dotted line* represents the LOD. Mann–Whitney: */**indicates a significant difference compared to pcDNA3 group at days 2 or 4 **(A, B)** or compared to mock **(D)**. BALF, bronchoalveolar lavage fluid; RSV, respiratory syncytial virus.

A preliminary test revealed that the minimal serum dilution that did not interfere with spiked-in IFN-λ detection was 16-fold for the bioassay and 5-fold for the ELISA (Fig. 5C, D) with both the Fawa-λ-luc and ELISA assays. IFN-λ was clearly detected at day 2 and 4 in the serum of mice that were electroinjected with the IFN-λ3 expressing plasmid, as well as at day 3 postnorovirus infection (Fig. 6A, B). At day 2 after a single IFN-λ3 injection, the cytokine was detectable in 1 out of 4 mice by ELISA and in 3 out of 4 mice by the bioassay. The detection limit was higher for the ELISA (194 pg/mL) than for the bioassay (9.29 pg/mL), showing the higher sensitivity of the luciferase-based bioassay (Fig. 6A, B).

Next, we compared IFN-λ detection by the Fawa-λ-luc assay and by ELISA in BALF of BALB/c mice that were mock infected or infected with RSV. BALF and lungs were collected 5 days postinfection. Plaque assay revealed that BALF of all infected mice contained between 1.8 × 10^2^ and 7.33 × 10^2^ PFU/mL of live replicating virus. The used UV irradiation procedure proved to readily inactivate RSV (RSV titer reduction of more than 46,000-fold; 4.6 × 10^4^ PFU to <1 PFU). Five-fold diluted control BALF did not modify IFN-λ2/3 detection in any of the 2 assays. Using 5-fold dilutions of the BALF as in the bioassay, we failed to detect any IFN-λ by ELISA (Fig. 6C). In contrast (Fig. 6D), IFN-λ was detected by Fawa-λ-luc assay at a very low concentration in the BALF of the RSV infected mice (5/6) and not detected in the control group.

We conclude that the Fawa-λ-luc-based assay described in this study allows to quantify IFN-λ from biological samples in a highly sensitive way.

## Discussion

We reexamined the species specificity of IFN-λ and highlighted a previously overlooked difference in bioactivity between mouse and human type III IFNs. Mouse IFN-λ3 displayed a surprising species specificity, with a 50-fold difference in relative antiviral activity when applied to LKR10 (mouse) and A549 (human) cells. This contrasted strongly with the closely related mouse IFN-λ2 (93% amino acid sequence identity), which displayed little species specificity and was 25 times more active in human cells than mouse IFN-λ3.

In contrast, human IFN-λ4 displayed a strikingly strong activity on mouse cells compared to human IFN-λ3. Although IFN-λ4 is not expressed in mouse and rat, it is highly conserved in many mammalian species (Key and others 2014) where it was shown to be cross-reactive. The nonhuman IFN-λ4 orthologs were reported to activate IFNLR1 in human cells, sometimes with a higher efficacy than human IFN-λ4 (eg, dog IFN-λ4) (Paquin and others 2016). The mouse cell response to human IFN-λ4 is thus in line with the ability of IFN-λ4 to be cross-reactive among mammalian species, although we do not have any physiological explanation for this phenomenon.

**FIG. 7.**
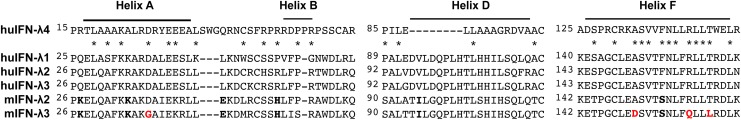
IFN-λ sequence alignments. Sequences of human and mouse IFN-λ regions implicated in receptor binding were aligned. Key amino acid residues involved in receptor activation that differs between mouse and human IFN-λ2 and IFN-λ3 sequences are indicated in *bold* in mouse sequences. Residues unique to mouse IFN-λ3 in helices A and F are indicated in *bold red*. *Indicates identical amino acids between human IFN-λ3 and IFN-λ4. Color images available online at www.liebertpub.com/jir

IFN-λ proteins follow the typical class II cytokine structure, consisting of 6 secondary structure elements (A–F) (Pestka and others 2004). Helix A is involved in both IFNLR1 and IL10Rβ binding, while helix D binds IL10Rβ and helix F binds IFNLR1 (Gad and others 2010). Among amino acid residues that have been characterized as crucial for IFNLR1 binding and activation (Gad and others 2010), a few divergences are observed between sequences from mouse and human IFN-λ (Fig. 7).

Notably, the more species-specific mouse IFN-λ3 has 4 amino acid residues in α helices A and F that diverge from the 5 other IFN-λ sequences, including the related mouse IFN-λ2. In helix A, Gly37 uniquely replaces the Asp residue present in the other sequences. In helix F, 3 residues are unique to mouse IFN-λ3: Asp 150 (Ala → Asp), Gln158 (Arg → Gln), and Leu161 (Thr → Leu). Future site-directed mutagenesis studies could help defining the key residues involved in the species specificity.

We designed a highly sensitive bioassay named “Fawa-λ-luc” based on luciferase reporter cells, specific for IFN-λ detection and quantification. The bioassay is based on a cell line that is naturally responsive to IFN-λ in which the type I IFN receptor gene was inactivated. Overexpression of the mouse IFNLR1 receptor in those cells led to increased induction of the reporter gene after IFN-λ treatment.

Unlike previously described bioassays, such as HL-116 fibroblasts stably transfected with the human IFNLR1 (Uze and Monneron 2007), Fawa-λ-luc cells permit the selective detection of type III IFN because they lack the type I IFN receptor. They also fail to respond to IFN-γ concentrations that are reached in infected mouse serum. This novel bioassay is efficient in measuring IFN-λ from mouse BAL and serum.

IFN-λ activity was hardly affected by UV exposure at virucidal doses, thus allowing detection in infected biological fluids. The high sensitivity of the assay permits sparing biological material as 10-fold more diluted serum samples could be used for bioassay detection (50–100-fold dilution) compared to the ELISA (5–10-fold). When cell toxicity of the tested samples is suspected, cell viability may independently be confirmed with nonlytic viability assays such as Resazurin-based tests, which are compatible with luciferase detection. Importantly, our bioassay offers a physiologic analysis, attesting of the functionality of the IFN-λ present in the samples, irrespective of specific activity.

The bioassay also allows to detect human IFN-λ. Given the divergent cross-species activity and the higher UV lability of the human type III IFNs, the bioassay might be used for individual subtype analysis, but would not fit for their detection in complex biological samples. Finally, it offers a sensitive detection of the divergent IFN-λ4, for which no efficient commercial test is available.
